# Cognitive functioning, depression, and HIV medication adherence in India: a randomized pilot trial

**DOI:** 10.1080/21642850.2014.913487

**Published:** 2014-05-05

**Authors:** Ryan Cook, Drenna Waldrop-Valverde, Aman Sharma, Szonja Vamos, Biraaj Mahajan, Stephen M. Weiss, Mahendra Kumar, Ritu Nehra, Deborah L. Jones

**Affiliations:** ^a^ Department of Psychiatry and Behavioral Sciences, University of Miami Miller School of Medicine, 1400 NW 10th Ave Suite 404A, Miami, FL33136, USA; ^b^ Nell Hodgson Woodruff School of Nursing, Emory University, 1520 Clifton Rd NE, Atlanta, GA30322, USA; ^c^ Post-Graduate Institute of Medical Education and Research, Chandigarh, India

**Keywords:** HIV, adherence, cognitive functioning, depression, India

## Abstract

*Objective*: India is home to the third-largest number of people living with HIV in the world, and no-cost antiretroviral therapy (ART) has been available across the country since 2004. However, rates of adherence to ART are often subpar in India, and interventions to increase adherence are warranted. Cognitive impairment and depression have been associated with ART non-adherence, and may also limit the impact of behavioral interventions designed to improve adherence. Studies have not evaluated the impact of cognitive impairment and depression on response to adherence interventions in India. *Methods*: Individuals new to ART (≤12 months prescribed) were recruited from a public hospital in Chandigarh, India. Participants (*N* = 80) were randomized to either a group medication adherence intervention (MAI) or an enhanced standard of care (ESOC) condition. The MAI consisted of three monthly gender-concordant group cognitive-behavioral sessions addressing HIV and ART, adherence, and HIV-related coping and social support. Participants were assessed at baseline for depression and cognitive functioning, and assessed monthly for adherence by pill count. *Results*: Adherence among participants receiving the MAI improved by about one day's dose over the course of the study, and no improvement was noted among those in the ESOC. Additionally, high rates of cognitive impairment (57%) and depression (25%) were identified among participants. There was no evidence that cognitive impairment moderated response to the intervention. However, while non-depressed participants benefitted from the intervention, depressed participants failed to show the same improvement. *Conclusions*: Results of this pilot study suggest that group behavioral interventions can be an effective strategy to promote ART adherence in this population, even among those demonstrating cognitive impairment. However, because of the negative impact of depression on adherence, future studies should continue to develop strategies to identify and treat it among people living with HIV in India.

## 1. Background

India is home to the third-largest number of people living with HIV in the world (UNICEF, 2008), with a countrywide HIV prevalence of nearly two and a half million people (UNGASS, 2010). Despite the fact that no-cost antiretroviral therapy (ART) has been nationally available since 2004, structural, individual, and cultural barriers have impeded widespread uptake, and it is estimated that only 425,000 (30–38% coverage) of those eligible are receiving antiretrovirals (ARVs) (WHO, UNAIDS, & UNICEF, 2011). As the distribution of no-cost ART has more recently been “decentralized” through the network of Community Care Centers, the number of those receiving ART is expected to rise (Shet et al., 2011).

Achievement of viral suppression requires sustained high levels of ART adherence (e.g. approximately 80–95% of doses taken as prescribed; Goldman et al., 2008; Nachega et al., 2007), and a recent systematic review and meta-analysis of ART adherence in India found a pooled adherence rate of only 70% (95% CI = 59–81%; Mhaskar et al., 2013). Surprisingly few interventions to improve ART adherence have been developed and tested in Indian populations; however, results have been encouraging. For example, Nyamathi, Hanson, et al. (2012) found that adherence improved after an intervention for rural Indian women utilizing local Accredited Social Health Activists (“Ashas”). Rodrigues et al. (2012) found that an intervention utilizing mobile-phone reminders improved adherence in a sample of South Indians. Additional interventions to improve adherence are warranted in this population.

Neurocognitive impairment (i.e. slowing of processing and fine motor speed along with difficulty with verbal learning, memory, and language; Lawler et al., 2011) has been identified as one of the earliest symptoms of HIV (Goodkin et al., 1997), is detectable prior to the onset of medication intervention (Cook et al., 2013), and is associated with a nadir CD4 count (Heaton et al., 2011). HIV-associated neurocognitive deficits have also been identified in 69% of aviremic patients following ART intervention, and in 64% of patients reporting no self-perceived deficits (Simioni et al., 2010). Cognitive impairment has been associated with non-adherence to ART among Indian populations (Anuradha et al., 2013). In fact, the relationship between medication adherence and neurocognitive functioning is complementary; suboptimal medication adherence may result in increased neurocognitive impairment, and individuals with impairment may be less likely to be adherent to medication (Andrade et al., 2012) as well as less likely to respond to interventions to improve treatment outcomes (Anand, Springer, Copenhaver, & Altice, 2010).

Similarly, the prevalence of depression is high among HIV-infected Indian individuals (Jagannath et al., 2011) and has been associated with non-adherence in India (De & Dalui, 2012; Nyamathi, Salem, et al., 2012). Individual and cultural factors, such as stigma, isolation, symptoms of illness, and psychological distress (Gonzalez, Batchelder, Psaros, & Safren, 2011; Nyamathi et al., 2010; Zelaya et al., 2012) may contribute to non-adherence and depression. Thus, despite the fact that behavioral interventions to improve ART adherence have achieved positive results in the Indian context, the efficacy of interventions may depend on the cognitive abilities and depressive symptomology of patients who undergo them. Currently, studies have not explored the relationship between depression, cognitive impairment, and response to an intervention to improve adherence in India. Additionally, although cognitive-behavioral interventions have been utilized to promote a number of positive health outcomes in a variety of settings (Brown & Vanable, 2008; Jones et al., 2013; Safren et al., 2009), cognitive-behavioral interventions have not been utilized to enhance adherence in India.

Given the subpar rates of ART adherence and the paucity of interventions targeting adherence in India, this study sought to pilot test a group cognitive-behavioral intervention to improve medication adherence among a Northern Indian sample. This study enrolled patients who were new to ART (≤12 months using ART) with a history of non-adherence, as they may be highly motivated to change behavior at the onset of medication use and thus may be highly likely to benefit from an intervention. It was hypothesized that participants receiving the group intervention would improve their medication adherence as compared to those receiving an individual enhanced standard of care (ESOC). Additionally, this study aimed to identify the prevalence of neurocognitive impairment and depression in this sample and examine those factors as potential moderators of participants' response to the intervention.

## 2. Methods

Prior to study onset, Institutional Review Board (IRB) approval was obtained from the University of Miami IRB. Ethical Review Committee approval was obtained from the Post Graduate Institution for Medical Education and Research (PGIMER), the Indian Council of Medical Research, and the National AIDS Control Organization.

### 2.1. Procedures

Eighty participants were recruited from October 2009 to September 2010 from the PGIMER Immunodeficiency Clinic in Chandigarh, India. Candidates were identified by clinic staff as non-adherent based on medication refill history and self-report, and screened to determine eligibility. Eligible participants were at least 18 years of age, HIV seropositive, and new to ART (3–12 months of ART medication use without a past ART prescription, verified by pharmacy records). Women who had previously taken nevirapine during pregnancy, persons reporting active alcohol or drug dependence, and inpatient hospital patients, those under hospice care, or those deemed unable to attend appointments or sessions due to profound illness or mental disability, were ineligible. Study offices were located at PGIMER in rooms separate from the clinic to reduce the potential for perceived coercion or influence by association with clinic services. Enrollment was carried out by study recruiters; clinic employees were not utilized for recruitment, assessment, or intervention activities. All study materials, including enrollment, informed consent, intervention, and assessment documents were administered in the local languages, Hindi and Punjabi. Prior to administration, items were translated and back-translated from English to Hindi and Punjabi and reviewed to ensure accuracy. Assessments were read to participants to remove limitations associated with literacy.

Following enrollment and provision of informed consent, participants were randomly assigned by the study coordinator to one of two time-matched treatment conditions, i.e. group medication adherence intervention (MAI) or individual ESOC, and followed for 6 months. Randomization was implemented using a table of random numbers generated by the principal investigator; randomization was unrestricted and participants were not allocated in any specific ratio (i.e. balance between conditions was not ensured). The sample size of 80 participants in this pilot study was determined based on the results of previous adherence research by the principal investigator.

### 2.2. Intervention

Prior to initiating the intervention, formative work was conducted to identify patient, provider, illness, contextual and cultural factors that influence medication adherence in the local Northern Indian context. Key informant interviews with HIV-positive patients and healthcare providers were conducted to assess the cultural acceptability and appropriateness of study measures and elements of the intervention. Additionally, focus group discussions were held with patients and providers (separately) in order to address issues related to HIV treatment, including access to care, medication adherence, retention in care, and HIV prevention. Patient focus groups were held in gender-specific groups to ensure comfort in discussion of sensitive topics, and this format was retained for the intervention due to high levels of acceptability.

#### 2.2.1. Group MAI content

Participants in the MAI condition attended regularly scheduled provider visits (monthly) plus three facilitator-led, hour-long, group sessions (1 per month for 3 months) addressing HIV and ART, adherence, and HIV-related coping and social support. The intervention utilized the Information Motivation Behavioral Skills Model (IMB; Fisher, Fisher, Amico, & Harman, 2006), providing information on ART and HIV (e.g. side effects, the relationship between treatment and disease progression) intended to increase motivation and skills (e.g. patient-provider communication, coping with side effects, pill reminders) to engage in and maintain treatment. This leads to positive health outcomes that provide additional motivation to maintain adherence over time (e.g. viral load suppression, perceptions of improved health, reduced distress). Sessions were manualized and utilized an interactive, gender-concordant group format (*n* ∼ 10 per group) with gender-matched facilitators to maximize the number of participants reached and the impact of facilitator and peer support. Facilitators were Masters level psychologists trained in administration of the intervention.

The initial MAI session primarily addressed knowledge about HIV and HIV-related medication, including the purpose of medication, consequences of non-compliance, and myths and misconceptions about HIV. In addition, participants were asked to assess current adherence, identify barriers to adherence and discuss concerns they may have had about their medication. During sessions two and three, participants were encouraged to share and solve problems related to adherence with peers and the facilitator. These sessions were designed to motivate and guide participants to develop a plan to maintain adherence, to identify barriers to adherence, and generate targeted strategies to overcome current and prospective barriers. During all sessions, participants were taught behavioral skills such as reframing attitudes and misconceptions about adherence, and were encouraged to brainstorm and role-play questions for upcoming physician visits.

#### 2.2.2. Individual ESOC content

Participants in the individual condition attended regularly scheduled provider visits plus three monthly time-matched individual sessions. Participants were shown a different HIV-educational video on healthy living each session (e.g. nutrition, exercise, relaxation).

### 2.3. Measures

#### 2.3.1. Cognitive functioning

Cognitive impairment at study baseline was assessed using a battery of three validated neuropsychological measures assessing specific domains of functioning including memory, psychomotor skills, and executive function. These domains were chosen because previous research has shown that they are likely to be affected by HIV and related to adherence (Lovejoy & Suhr, 2009; Waldrop-Valverde et al., 2006). Measures included the Hopkins Verbal Learning Test-Revised (HVLT-R), the Grooved Pegboard Test, and the Color Trails Test (CTT). Normative scores on these measures were obtained from a Southern Indian sample without HIV or other neuropsychological disorders (Yepthomi et al., 2006); the normative sample was similar to study participants in this study in sex, age, and education. Z-scores were calculated for the Hopkins total recall score (trial 1 + trial 2 + trial 3), the Hopkins delayed recall score, time to complete the Grooved Pegboard Test using the non-dominant hand, and time to complete Color Trails 1 and 2. “Time to complete” scores were reversed, so that increased time would result in a lower Z-score. Participants were classified as cognitively impaired if they scored at least 1 standard deviation below the mean (*Z* ≤ 1) on any two of the neuropsychological tests at baseline (Antinori et al., 2007).

The HVLT-R (Benedict, Schretlen, Groninger, & Brandt, 1998) contained two measures assessing short-term learning and memory performance. Participants were read a set of 12 words and asked to repeat the words immediately afterwards. The first measure, total recall, was the sum of items correctly recalled after three trials (maximum score = 36). Delayed recall of the set of words was assessed 20 min following the third trial; the delayed recall score is the total number of correct responses (maximum score = 12). The HVLT-R has demonstrated high construct validity in HIV-positive populations (Woods et al., 2005).

The Grooved Pegboard Test (Klove, 1963) utilized a small pegboard containing a 5 × 5 set of slotted holes angled in different directions. Each metal peg had a ridge along one side requiring it to be rotated into position for correct insertion. The score was time to completion. The test was given using first the dominant hand, then the non-dominant hand. Time to completion using the non-dominant hand was utilized for analyses because of its high predictive power for cognitive impairment (Carey et al., 2004). The Grooved Pegboard Test has demonstrated high reliability (Ruff & Parker, 1993).

The CTT (Maj et al., 1993) consisted of two exercises: test 1 measured processing speed by timing test-takers to connect numbered circles in order and test 2 consisted of alternately colored circled numbers used to measure speed of attention, sequencing, mental flexibility, and visual search and motor functions. The CTT was developed by the World Health Organization to be culturally unbiased and has been widely used among HIV-positive individuals. High sensitivity of the CTT to detect cognitive impairment in HIV-positive individuals in different cultures has been established (Maj et al., 1993).

#### 2.3.2. Depression

The Beck Depression Inventory II (BDI) (Beck, Steer, Ball, & Ranieri, 1996) was administered to assess for depression at baseline. Participants meeting the criteria for at least mild depression were classified as depressed (total BDI score of 14 or higher). The BDI has demonstrated high reliability in Indian populations (*α* = 0.96; Basker, Moses, Russell, & Russell, 2007).

#### 2.3.3. Adherence

Pill count adherence was measured each month in person by an assessor and evaluated using the prescribed dosage and pharmacy fill records. An adherence score was derived, representing the absolute value of the difference between the number of pills remaining and number expected to remain (representing 100% adherence), with a tolerance of one day's dosage to account for the time of day the pill count was conducted. A score of zero indicated perfect adherence; smaller values indicated better adherence.

The adherence score was adjusted each month to incorporate the previous months' remaining pills, ensuring that the length of observation time was the same between each assessment of adherence. Because the first month's pill count could not be adjusted and thus reflected an unknown length of observation time prior to study entry, baseline adherence scores were computed from month two, reflecting adherence during the first month of study participation, including MAI/ESOC session 1. Baseline adherence was compared to adherence during month six, which was the final assessment.

### 2.4. Statistical analyses

Descriptive statistics are provided for demographics, cognitive test scores, and baseline adherence values. Bivariate analyses (e.g. *t* tests) were conducted to test for differences between MAI and ESOC conditions in demographic and outcome variables at baseline. In order to analyze adherence over time, paired samples *t* tests were planned. However, the assumption of normality was significantly violated, and the moderate sample size limited the robustness of the analysis. Therefore, in order to satisfy the distributional assumptions of the outcome variable (i.e. pill count) a repeated measures negative binomial regression model was conducted using SAS PROC GLIMMIX (N.B.: a Poisson model was also fit and significant overdispersion was noted, thus a negative binomial regression was chosen as the appropriate analysis). Pill count was the outcome, and predictors included time (baseline, study completion), condition (MAI vs. ESOC), and the interaction between time and condition. To estimate the effects of the intervention over time, planned comparisons between groups at each timepoint and between timepoints within each group were conducted. To test the potential moderating effects of cognitive impairment and depression, these terms were added to a model including only those participants in the MAI condition. Adherence over time was compared between cognitively impaired vs. cognitively unimpaired and depressed vs. non-depressed participants. All analyses were completed using SAS v.9.3 (SAS Institute, Cary, NC) using a two-tailed level of significance of *α* = 0.05.

## 3. Results

### 3.1. Characteristics

Participants (*n* = 80) were, on average, 38 years of age and were predominantly male (*n* = 56, 70%). More than half (*n* = 46, 58%) reported at most 9 years of education, however, most were employed (*n* = 58, 73%) and earned an average monthly income of 4346 ± 4640 Indian Rupees (∼US$80). Over three quarters were married (*n* = 63, 78%) and 62% (*n* = 50) lived in a rural setting. The mean time since HIV diagnosis was 18.2 ± 24.6 months, and the mean time on ARVs was 6.9 ± 3.0 months. Randomization resulted in 34 participants assigned to the MAI condition and 46 to the ESOC condition. Attendance at the study sessions averaged 91% and did not differ between conditions. There were no differences in baseline demographic variables between participants in the MAI and ESOC conditions. Demographic information by condition is presented in Table 1.

**Table 1.** Demographics.

Characteristic	MAI (*n* = 34) (*n*, %)	ESOC (*n* = 46) (*n*, %)	*χ*^2^(1 df), *p*
*Gender*			1.44,^a^ .23
Male	21 (62%)	35 (76%)	
Female	12 (35%)	11 (24%)	
Hijra (transgender)	1 (3%)	0 (0%)	
*Employment status*			0.11, .74
Employed	24 (71%)	34 (74%)	
Unemployed	10 (29%)	12 (26%)	
*Years of education*			0.04, .84
≤9 years	20 (59%)	26 (56%)	
≥10 years	14 (41%)	20 (44%)	
*Living area*			1.65, .20
Urban	10 (29%)	20 (43%)	
Rural	24 (71%)	26 (57%)	
*Marital status*			0.18, .67
Married	26 (76%)	37 (80%)	
Single/separated/widowed	8 (24%)	9 (20%)	
	*m* (sd)	*m* (sd)	*t*(78 df), *p*
Monthly income (Indian rupees)	5373.5 (5840.8)	3586.9 (3377.4)	1.6, .12
Age	39.4 (8.9)	37.3 (7.9)	1.12, .27
Time since HIV diagnosis (months)	20.6 (28.6)	16.5 (21.4)	0.72, .47
Time on ARVs (months)	7.1 (3.2)	6.8 (2.8)	0.42, .68

^a^Due to small cell size, *χ* ^2^ value was computed excluding a single Hijra (transgender male) participant.

### 3.2. Adherence

Baseline unadjusted adherence scores ranged from 0 to 23, averaging 2.83 ± 4.02; there was no difference between MAI and ESOC condition participants in unadjusted adherence at baseline [*t*(38.4 df) = 1.19, *p* = .24]. Participants were asked if they had discarded any pills prior to assessment. One ESOC condition participant (1% of the sample) reported throwing away pills at both baseline and follow-up, and all other participants reported never throwing away pills at any time. The analysis was run both including and excluding this participant and results were similar; the results are presented excluding this participant. A repeated measures negative binomial regression was conducted to examine adherence over time, and predicted means were compared between groups at each timepoint and between timepoints in each group. Comparisons revealed that adherence among MAI participants improved over the duration of the study [*t*(89.2 df) = 2.63, *p* = .01] and adherence among ESOC participants did not change [*t*(78.9 df) = 0.45, *p* = .65]. Adherence did not differ between groups at either timepoint [baseline, *t*(72.4 df) = 0.74, *p* = .46; follow-up, *t*(82.6 df) = 1.37, *p* = .18]. The results of this analysis along with predicted group mean adherence scores and 95% confidence intervals for each timepoint and condition are presented in Table 2.

**Table 2.** Negative binomial regression of adherence^a^ by condition over time.

Parameter	*b* (se), *p*		95% CI (*b*)
Condition (ESOC vs. MAI)	0.643 (.440), .149		(−0.234, 1.519)
Time (baseline vs. follow-up)	**0.950 (.361), .010**		**(0.232, 1.669)**
Condition × time	−0.828 (.463), .077		(−1.749, 0.093)
*Between- and within-group comparisons*			
	MAI condition mean (95% CI)	ESOC condition mean (95% CI)	Difference between groups (95% CI)
Follow-up (month 6)	0.87 (0.44, 1.74)	1.66 (0.96, 2.87)	−0.64 (−1.52, 0.23)
Baseline (month 2)	2.26 (1.46, 3.49)	1.88 (1.28, 2.74)	0.18 (−0.39, 0.76)
Difference within groups (95% CI)	−**0.95 (**−**1.67,** −**0.23)**	−0.13 (−0.70, 0.46)	

Note: Bold indicates statistical significance.

^a^Adherence score = absolute value of the difference between the number of pills remaining and the number of pills expected to remain, with a tolerance of one day's dose. Zero indicates perfect adherence; smaller values indicate better adherence.

The most common cART prescriptions were for Duovir-N^®^ (lamivudine/zidovudine/nevirapine; *n* = 37 prescribed) or Trimune^®^ (stavudine/lamivudine/nevirapine; *n* = 24). Seventeen participants were also prescribed efavirenz, and one participant was prescribed a cART therapy regimen which included a protease inhibitor (lopinavir/ritonavir). Participants were grouped into those taking additional medications (i.e. efavirenz or lopinavir/ritonavir) and those not taking additional medications, and adherence was examined over time; regimen complexity did not impact adherence.

### 3.3. Cognitive functioning and depression

Participants scoring ≤1 standard deviation below the normative mean on any two tests in the cognitive battery were classified as cognitively impaired, resulting in more than half of participants classified as cognitively impaired (*n* = 46, 57%; 95% CI = 47–68%). The proportion of cognitively impaired participants did not differ between conditions (53% of MAI participants (*n* = 18) vs. 61% of ESOC condition (*n* = 28), *χ* ^2^ (1 df)  = 0.69, *p* = .41), nor was there any difference between conditions on any single test in the battery (see Table 3). Normative and sample means and standard deviations as well as *Z* scores for cognitive functioning tests are presented in Table 3.

**Table 3.** Cognitive battery scores at baseline.

Test (baseline)	Normative^a^*m* (sd)	ESOC condition *m* (sd), *Z*	MAI condition *m* (sd), *Z*	*t*(78 df), *p*^b^
Verbal learning total	21.7 (5.2)	20.07 (5.4), −0.31	20.29 (5.1), −0.27	0.19, .84
Verbal memory total	8.2 (2.1)	7.02 (2.5), −0.56	7.24 (2.3), −0.46	0.39, .70
Pegboard, non-dominant	81.9 (13.8)	103.39 (37.0), −1.56	115.76 (47.8), −2.49	1.30, .20
Color trails 1	59.8 (20.5)	79.14 (37.4), −0.94	92.45 (49.3), −1.59	1.37, .17
Color trails 2	138.5 (50.1)	192.77 (105.53), −1.08	211.25 (105.7), −1.45	0.77, .44

^a^Normative data drawn from Yepthomi et al. (2006).

^b^ *t*-Statistics and associated *p* values represent comparisons between ESOC and MAI groups.

Baseline depression scores ranged from 0 to 38, averaging 9.39 ± 8.58. Participants scoring ≥14 on the BDI at baseline met the criteria for at least mild depression; one quarter of participants met this criteria (*n* = 20, 25%; 95% CI = 16–34%). The proportion of depressed participants at baseline did not differ between conditions (26% of MAI condition (*n* = 9) vs. 24% of ESOC participants (*n* = 11), *χ* ^2^ (1 df) = 0.04, *p* = .84). Additionally, there was no association between depression and cognitive impairment (*χ* ^2^ (1 df) = 0.11, *p* = .73).

In order to test the potential moderating effects of cognitive impairment and depression, adherence over time was compared between cognitively impaired vs. cognitively unimpaired and depressed vs. non-depressed participants in the MAI condition. There was no difference in unadjusted adherence at baseline between cognitively impaired and unimpaired participants (*t* (26.06 df) = 0.90, *p* = .38) or depressed and non-depressed participants (*t* (10.13 df) = 0.46, *p* = .66). There was no evidence that adherence over time differed between cognitively impaired and cognitively unimpaired participants; a significant and very similar improvement in adherence was noted in both groups (see Table 4). However, there was evidence that depression moderated response to the intervention. Non-depressed participants improved their adherence over time, however, the improvement among depressed participants was smaller and not statistically significant (see Table 5).

**Table 4.** Moderation analysis of cognitive impairment on adherence^a^ over time within MAI participants.

Parameter	*b* (se), *p*		95% CI (*b*)
Cognitive impairment (unimpaired vs. impaired)	−0.261 (.518), .616		(−1.299, 0.776)
Time (baseline vs. follow-up)	**0.815 (.303), .015**		**(0.177, 1.453)**
Cognitive impairment × time	−0.017 (.474), .972		(−0.995, 0.962)
*Between- and within-group comparisons*			
	Cognitively impaired mean (95% CI)	Cognitively unimpaired mean (95% CI)	Difference between groups (95% CI)
Baseline (month 2)	2.52 (1.37, 4.65)	1.91 (0.93, 3.91)	0.28 (−0.67, 1.22)
Follow-up (month 6)	1.12 (0.56, 2.24)	0.86 (0.40, 1.86)	0.26 (−0.78, 1.30)
Difference within groups (95% CI)	−**0.81 (**−**1.45,** −**0.18)**	−**0.80 (**−**1.54,** −**0.06)**	

Note: Bold indicates statistical significance.

^a^Adherence score = absolute value of the difference between the number of pills remaining and the number of pills expected to remain, with a tolerance of one day's dose. Zero indicates perfect adherence; smaller values indicate better adherence.

**Table 5.** Moderation analysis of depression on adherence^a^ over time within MAI participants.

Parameter	*b* (se), *p*		95% CI (*b*)
Depression (non-depressed vs. depressed)	−0.772 (.563), .177		(−1.905, 0.361)
Time (baseline vs. follow-up)	0.307 (.391), .444		(−0.522, 1.135)
Depression × time	0.753 (.481), .133		(−0.249, 1.756)
*Between- and within-group comparisons*			
	Depressed mean (95% CI)	Non-depressed mean (95% CI)	Difference between groups (95% CI)
Baseline (month 2)	2.26 (0.94, 5.44)	2.22 (1.27, 3.90)	0.02 (−1.02, 1.06)
Follow-up (month 6)	1.67 (0.65, 4.28)	0.77 (0.41, 1.45)	0.77 (−0.36, 1.90)
Difference within groups (95% CI)	−0.31 (−1.14, 0.52)	−**1.06 (**−**1.63,** −**0.49)**	

Note: Bold indicates statistical significance.

^a^Adherence score = absolute value of the difference between the number of pills remaining and the number of pills expected to remain, with a tolerance of one day's dose. Zero indicates perfect adherence; smaller values indicate better adherence.

## 4. Discussion

This study sought to evaluate the impact of an IMB-based pilot group intervention utilizing cognitive-behavioral techniques to improve medication adherence, identify the prevalence of neurocognitive impairment and depression, and examine the potential for cognitive impairment and depression to moderate the effect of the intervention. Study outcomes illustrate that participants receiving the MAI improved their adherence, while the adherence of those receiving an ESOC did not change. The change in adherence observed in this study was modest, representing an increase of about one day's dose. However, studies have identified virological failure rates of 29% at adherence rates of 80–95% (Goldman et al., 2008) and 25% at adherence rates of 80–99% (Nachega et al., 2007). Thus, even a small increase in adherence may mean a significant improvement in virological outcomes. While first-line ART is readily available in India, many second-line regimens may be cost-prohibitive (Freedberg et al., 2007). Thus, it is vital for HIV-infected individuals to maintain high levels of adherence in order to avoid developing resistance to first-line therapy. Additionally, this study enrolled some participants who were adherent to their medication upon study entry. The “ceiling effect” of those participants may have diluted estimates of treatment effects for those who were highly non-adherent at study entry and demonstrated greater improvement.

Similar to previous studies in this population (Cook et al., 2013; Gupta et al., 2007; Jagannath et al., 2011), this study identified high rates of neurocognitive impairment and depression. In contrast with earlier studies (Anand et al., 2010), there was no evidence that cognitive impairment influenced the potential for participants to benefit from the intervention. While this finding highlights the utility of using a behavioral intervention to increase adherence among patients with cognitive impairment, it is important to note that the failure to find a difference in response to the intervention between impaired and unimpaired participants may have been due to the small sample size of this pilot study. Although well-established criteria were utilized to classify participants as cognitively impaired, this dichotomization likely resulted in low power to detect their impact on adherence. Future studies should include a larger sample in order to definitively conclude that cognitively impaired individuals can benefit from adherence interventions similarly to unimpaired individuals. While non-depressed participants benefitted from the intervention, depressed participants failed to show the same improvement. This finding is consistent with previous research (Thomas et al., 2011) and supports further efforts to identify and treat depression in this population.

In addition, the primary outcome of this study, pill count, has been criticized because of the potential for miscalculating adherence (e.g. patients may misrepresent adherence by discarding pills before assessments or adherence may be under/overestimated due to the time of day the count was administered; McMahon et al., 2011). However, the adherence calculation in this study included one day's tolerance to account for the time of assessment, and although electronic pill monitoring was not possible, 99% of the sample reported no discarding of pills. This study utilized a simple randomization strategy, which was easily implemented but resulted in an unequal number of participants in each condition. Unequal assignment to treatment groups generally results in a loss of statistical power; however, the loss is minimal until the ratio of group assignment is larger than about 3:1 (Pocock, 1995). Thus, it is unlikely that the unbalanced group size negatively impacted study power. Additionally, this study used measures that have been adopted in India, but were not developed for that population (e.g. the BDI). However, assessments were carefully translated and back-translated from English into the local languages and verified for accuracy. Finally, the use of normative data from a different region of India to assess cognitive functioning may have been suboptimal; however, data from Northern India were not available. Although the languages of administration differed between the two studies, the process of translating measures was similar between this study and the study providing normative data.

Although the improvement was modest, the results of this study suggest that a group intervention may improve adherence among those new to medication in India, including those with cognitive impairment. Widespread distribution of ART suggests that rapid, large-scale HIV adherence education interventions may be an important concomitant component of ART provision. Group interventions may also be a cost-effective strategy to provide HIV-related health behavior information to larger numbers of patients, including settings with patients with neurocognitive impairment. Research should continue to explore behavioral methods to introduce healthy behaviors early in disease management to establish and maintain long-term adherence.

## Funding

This grant was made possible by a grant from the National Institutes of Health, R21NR011131, and with the support of the Post Graduate Institute for Medical Education and Research.
